# Supplementary figures and images for: Mitochondrial DNA copy number, metabolic syndrome, and insulin sensitivity: Insights from the Sugar, Hypertension, and Physical Exercise studies
Source: PLoS One. 2022 Jul 18;17(7):e0270951. doi: 10.1371/journal.pone.0270951 (PMC9292076; doi:10.1371/journal.pone.0270951)

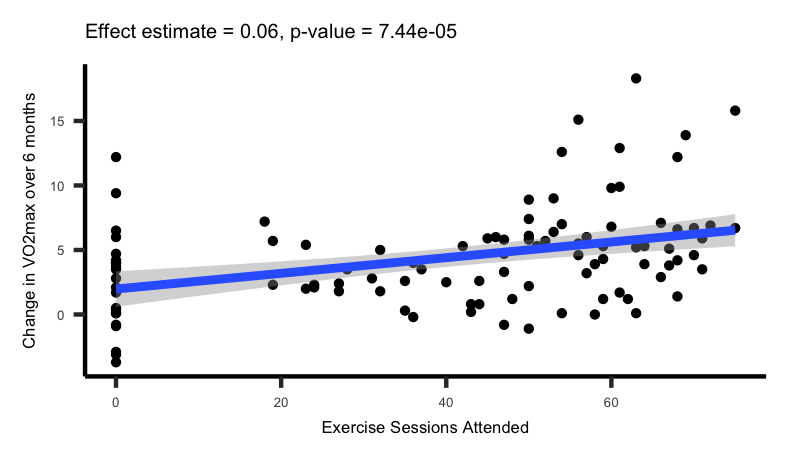

Supplement: S1 Fig — The number of exercise sessions attended is significantly associated with a 6-month increase in VO2max. (TIF) [file pone.0270951.s001.tif]

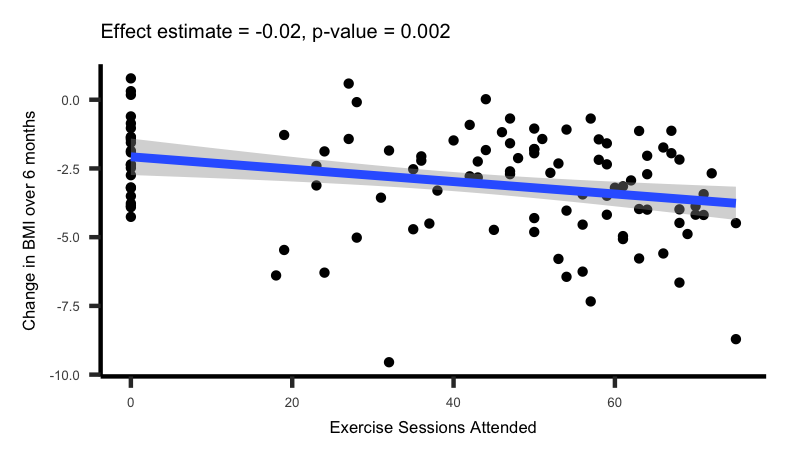

Supplement: S2 Fig — There was a significant association between a greater number of exercise sessions attended and decreased BMI over the 6-month intervention period. (TIF) [file pone.0270951.s002.tif]

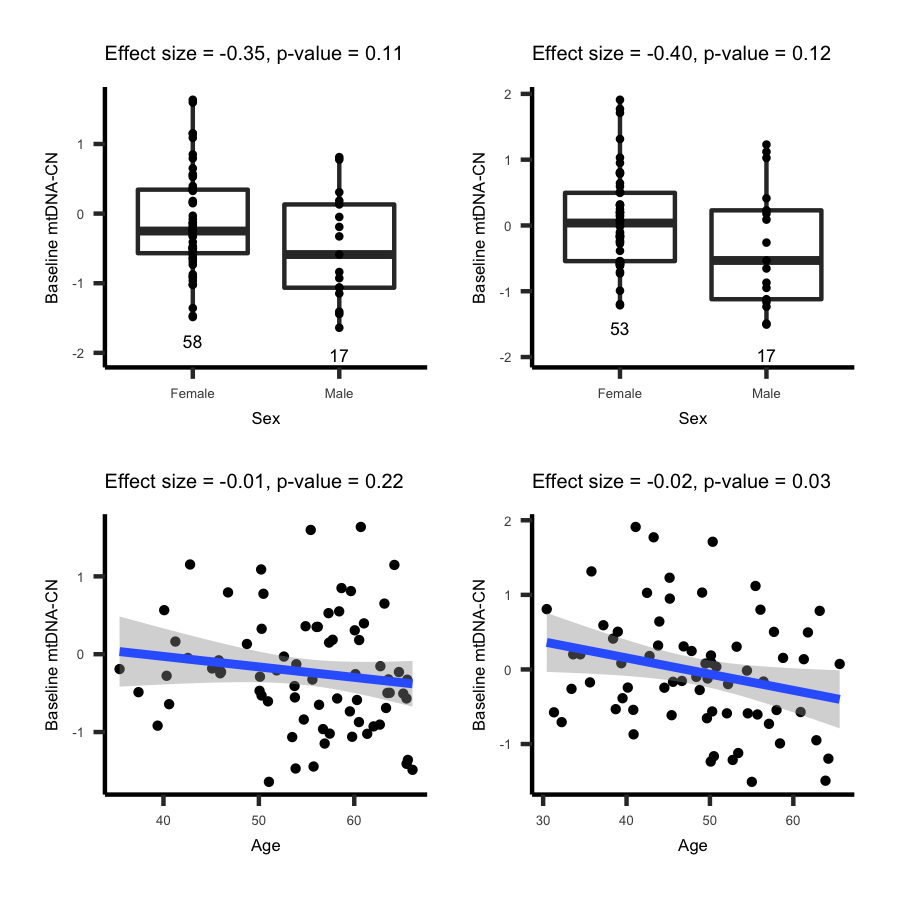

Supplement: S3 Fig — Associations between mtDNA-CN and age and sex are in the expected directions when stratifying by SHAPE study. SHAPE3 on the left, SHAPE5 on the right. (TIF) [file pone.0270951.s003.tif]

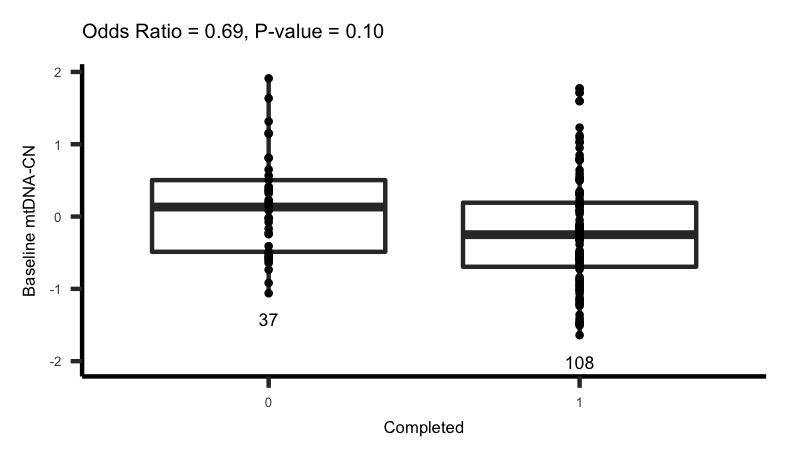

Supplement: S4 Fig — Individuals who dropped out of the study had significantly higher mtDNA-CN. (TIF) [file pone.0270951.s004.tif]

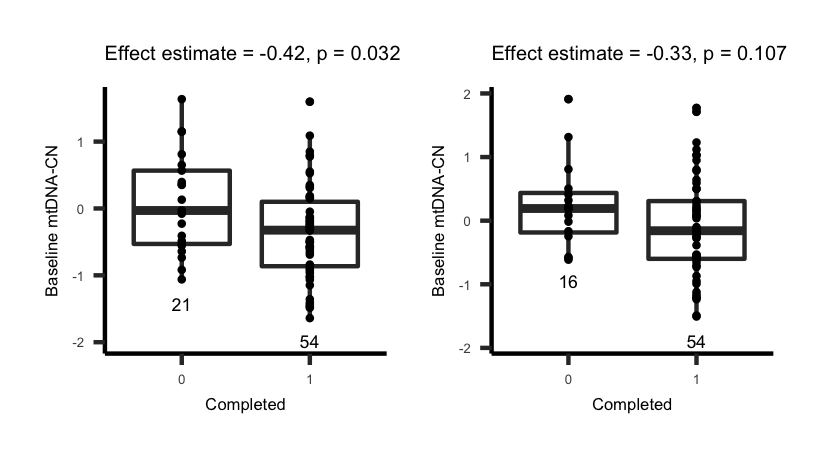

Supplement: S5 Fig — Individuals who dropped out of the study have significantly higher mtDNA-CN than those who were retained (On left, SHAPE3, on right, SHAPE5). (TIF) [file pone.0270951.s005.tif]

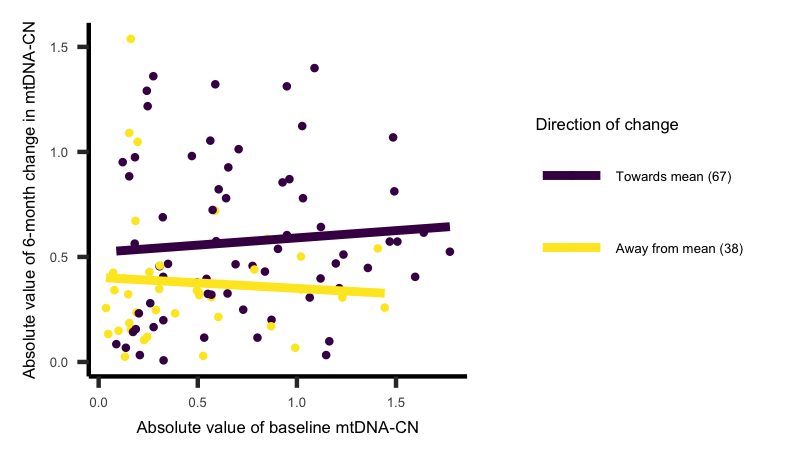

Supplement: S6 Fig — mtDNA-CN measured at baseline is associated with a change in mtDNA-CN in the direction of the mean. Purple points denote individuals with final mtDNA-CN measurements closer to the mean than their baseline measurements, while yellow is vice versa. Significantly more individuals move towards the mean (chi-squared p = 0.004). For the purple samples, absolute magnitude of baseline mtDNA-CN is positively correlated with the absolute value of 6-month change, however, this association is not significant (P = 0.51). (TIF) [file pone.0270951.s006.tif]

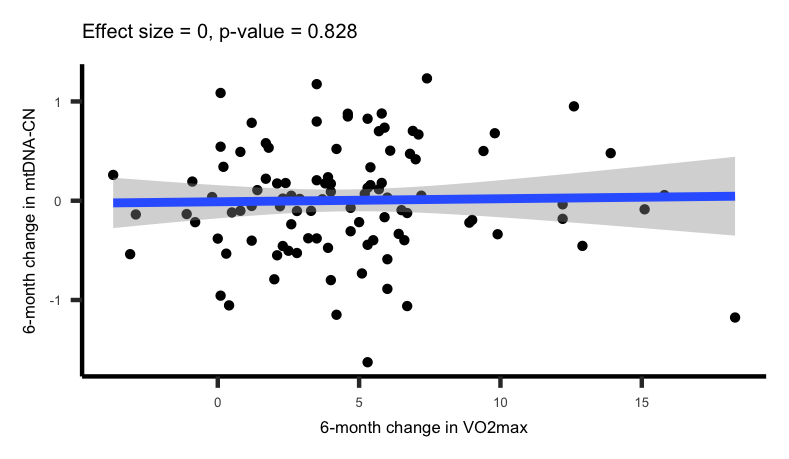

Supplement: S7 Fig — There was no significant association between 6-month change in VO2 max and 6-month change in mtDNA-CN. (TIF) [file pone.0270951.s007.tif]
